# The psychosocial implications of social distancing for people with COPD: some exploratory issues facing a uniquely marginalised group during the COVID-19 pandemic

**DOI:** 10.1057/s41285-021-00166-0

**Published:** 2021-06-14

**Authors:** Heather Yoeli

**Affiliations:** Department of Anthropology, Institute for Medical Humanities, University of Durham, Caedmon Building, Leazes Road, Durham, DH1 1SZ UK

**Keywords:** COPD, COVID-19, Health inequalities, Intersectionality, Racism, Stigma

## Abstract

Chronic obstructive pulmonary disease (COPD) is an illness associated with intersectional poverty and stigma in old age; people with COPD are susceptible to anxiety, loneliness and isolation. People with COPD who contract COVID-19 are at high risk of serious complications, intensive medical needs and death and are, therefore, required to socially distance particularly assiduously. This paper offers an embodied phenomenological analysis of the emerging theoretical literature exploring the impact of social distancing upon people with COPD. Firstly, people with COPD are aware of how respiratory illness feels, are anxious about COVID-19 and afraid of being denied care. Future research might consider how people with COPD may be susceptible to “coronaphobia” and mental health consequences of the pandemic. Secondly, COPD tends to affect older people within the most intersectionally marginalised socioeconomic groups. Future research should remain mindful that people with COPD may be among the most lonely and least able to access health and social care services online than others. Thirdly, pandemics are known to intensify pre-existing social stigmas. Researchers and practitioners alike should be conscious that people with COPD may become increasingly stigmatised, especially those from intersectionally disadvantaged minorities.

## COPD in the era of social distancing

Chronic obstructive pulmonary disease (COPD) is diagnosis which encompasses chronic bronchitis and emphysema, sometimes with elements of bronchiectasis and refractory asthma (Vestbo et al. 2013). Across the world, approximately 64 million people live with COPD, with 90% of these in the Global South (WHO 2019) and three million in the UK (DoH 2011).

People with COPD describe dyspnoea, or breathlessness, as the most distressing symptom of their illness. People with COPD often feel more breathless and more disabled by their breathlessness than measures of their lung function might predict, something that is generally attributed to the difficulties with interoceptive awareness which often accompany chronic illness (Macnaughton 2020). This breathlessness is frequently accompanied by anxiety, social isolation and loneliness; these arise both as a consequence of the stress of managing breathlessness itself and as a response to the socioeconomic isolation, exclusion and stigmatisation which co-exist with the illness (Williams and Carel 2018). Across the world, COPD is largely a disease of poverty and intersectional disadvantage among older adults. In the UK, health disparities related to the prevalence, diagnosis and prognosis for COPD are significantly more pronounced than those of most similarly affluent countries (DoH 2011).

Because COVID-19 attacks the respiratory system, people with COPD who contract the virus are thought to be particularly “at risk” of becoming acutely unwell, of needing hospital care and mechanical ventilation, and of death (gov.uk 2020; nhs.uk 2020). Within the UK, as within other global healthcare systems, people with COPD are regarded as extremely “vulnerable” and are therefore expected to adhere especially assiduously to social distancing procedures. Nevertheless, the socioeconomic deprivation, isolation and stigmatisation associated with the illness means that people with COPD and the challenges which social distancing poses to them, have hitherto been afforded little public health research or policy consideration.

The virulence and lethality of COVID-19 has arisen so unexpectedly and so rapidly that research undertaken during the pandemic’s first year has focussed primarily upon the most proximal imperatives of clinical treatment, infection control, and vaccine development. As such, there has to date been little empirical investigation of the lived experiences of specific groups of people who are living through the pandemic’s risks and restrictions without having been affected by its virus, though some researchers are now initiating such studies with reference to people with COPD (Burlacu et al. 2021; McAuley et al. 2021; Yoeli and Macnaughton 2021).

In order to inform and to direct the emergence of such research studies, this paper offers an overview of recent philosophical and theoretical innovations within the field of embodied phenomenology exploring the existential meanings of social distancing (Carel et al. 2020; Dolezal 2020; Goli 2020; Rossolatos 2020; Vallee 2020). In so doing, this paper seeks to apply these meanings to what is already known from phenomenological research about the embodied experiences of people with COPD (Williams and Carel 2018; Macnaughton 2020) to consider how people with COPD in the UK may experience social distancing.

This paper offers a brief overview of emerging phenomenological analyses of social distancing before identifying three specific challenges which people with COPD may encounter. This is therefore not an empirical review but instead an exploratory and largely polemical article, which draws upon Merleau-Ponty’s (1968) embodied phenomenology to make sense of what social distancing means for vulnerable bodies in a pandemic. In its speculation and polemic, this article aims to inspire and to inform future research into the COVID-era experiences of people in the UK with COPD.

## Phenomenologies of social distancing

### Social distancing as a phenomenon and a place

Throughout the history of pandemics and plagues, populations have understood the collective benefits of keeping infected individuals (or those deemed vulnerable to infection) away from others (Mackowiak and Sehdev 2002). Whenever a pandemic or plague has been recognised as airborne in transmission, populations have deduced collective benefits of keeping as many people as possible (or as many of possible perceived undesirables stigmatised as specific spreaders of the illness) away from public areas (Huremović 2019; Jones 2020). From the Black Death of the 1665-6 (Wallis 2006) to the H1N1 Influenza pandemic of 1918-9 (Markel et al. 2007), villages, towns and cities have implemented localised procedures for keeping people physically separated. At national and transnational levels, protocols for what had come to be referred to as “social distancing” (sometimes referred to as “physical distancing”) began during the SARS and Ebola outbreaks of the early 2000s (Barbisch et al. 2015).

During the early months of the COVID-19 pandemic, the UK government introduced social distancing as part of a strategy of “flattening the curve” (Ferguson et al. 2020) to safeguard overall health service capacity from being outpaced by the needs of infected patients requiring nursing and medical care, ICU beds and mechanical ventilation. Because individuals with pre-existing chronic medical conditions such as COPD were, if infected, more likely to become seriously ill, people with such conditions were deemed “vulnerable” and expected to adhere to more stringent levels of social distancing than others (gov.uk 2020; nhs.uk 2020).

Across the world, people with COPD and similarly “at risk” medical conditions are under pressure to embody social distancing not only for their own safety but for the overall wellbeing of society. Because of the social and ethical challenges that this complexities of social distancing has produced, Rossolatos (2020) asserts the importance of regarding social distancing not as an epidemiological or political construct or as a task to be implemented but as an ontological reality, as a place that exists to separate human beings from physical proximity to one another. Phenomenologies of social distancing identify two dimensions to this recently emergent place (Carel et al. 2020; Dolezal 2020). Social distancing exists as the physical spaces which people place between themselves and others in public and also as the online spaces on social media and video conferencing platforms that people use as alternatives to face-to-face encounters.

### Social distancing as a challenge to embodiment and intercorporeality

Through changing the nature of place, and through changing the nature of how humans relate and interact within this place, social distancing both face to face and online is changing and challenging the phenomenology of the human body as a social being. This invites new consideration of Heidegger’s ontology (Carel et al. 2020; Rossolatos 2020) and new thinking about concepts of intercorporeality (Dolezal 2020; Katila et al. 2020) and embodiment (Carel et al. 2020; Vallee 2020).

Within phenomenology, the concept of embodiment refers to the individual or collective experience of the human body, and upon the role of the body in the lived experience of being human. Building upon Heidegger’s (1927) account of human existence as a multi-faceted and socially mediated phenomenon, Merleau-Ponty (1968) emphasises that embodiment involves both being and having a body. These dual facets to embodiment become especially pertinent when disease or disability cause the specific aspects of a physical body to function less efficiently than, or differently from, others (Leder 1984; Csordas 1993). Notwithstanding the way that pandemics invariably lead to already-marginalised bodies being further shamed, stigmatised and scapegoated, COVID-19 emphasises of social implications of embodiment (Carel et al. 2020). Within a pandemic, however, anyone’s body could potentially become infected, breathless, and sick from COVID-19; anyone’s body and anyone themselves might embody disease and illness. Embodiment becomes fragile and changeable as our increasing reliance on electronic forms of communication leaves an increasingly complex “shimmer” of our data online (Vallee 2020).

The concept of intercorporeality (also called “carnal intersubjectivity”) originates specifically from the work of Merleau-Ponty (1968). Intercorporeality is the intersubjectivity (shared human experience) of simultaneously being and having a body, and the impact of this intersubjectivity upon human social interactions and relationships (Merleau-Ponty 1968). Intercorporeality involves a range of implicit and inherent bodily instincts and rituals in human interaction. Intercorporeality involves an inherent degree of trust between humans, a trust that is predicated upon their common experience of being human. Through the physical space that social distancing places between people, whether face to face or online, social distancing disrupts intercorporeality because it precludes the opportunities and dangers of proximity and touch (Dolezal 2020), preventing the unique ways that humans can both become intimate with and cause harm to the bodies of one another.

Social distancing thereby undermines the fundamentally inherent trust that humans place in one another, and specifically in the bodies of one another. In essence, social distancing undermines human trust of people whose bodies appear “malfunctioning” or “different”. This has particular implications for people living with chronic illnesses such as COPD that are already marginalised or stigmatised, especially when these people already belong to already disadvantaged socioeconomic or ethnic group. It is through this lens of Merleau-Ponty’s embodied phenomenology (1968) that this paper approaches its analysis.

## Three challenges facing people living through social distancing with COPD

### Social distancing with an awareness of clinical vulnerability: uncertainty, insecurity and coronaphobia

The extent to which social distancing disrupts fundamental aspects of embodiment, intercorporeality and trust, together with the wider economic and political upheavals caused by the pandemic, has led to a widespread atmosphere of global uncertainty experienced on both an emotional and social level (Carel et al. 2020). This is arguably especially the case for people living with COPD: they are told they are “at risk” and “vulnerable” to becoming seriously ill or die from COVID-19 (nhs.uk 2020); they have been subjected to the most stringent forms of social distancing (gov.uk 2020); they are uniquely aware of what breathlessness is and feels like (Philip et al. 2020; Askov Mousing and Sørensen 2021). Whereas people with a range of pre-existing illnesses and disabilities have expressed fears about not being prioritised for COVID-19 medical treatment, people with respiratory conditions are uniquely attuned to what this potential denial of treatment might involve, and to the discomfort and terror of how a breathless death from respiratory failure might feel (Philip et al. 2020). Emerging research suggests that the COVID-19 pandemic may significantly be harming the interoceptive abilities and mental resilience of people with long-term respiratory conditions (De Lucia et al. 2020), and is causing existential fear and anxietymost acutely among people with COPD (Askov Mousing and Sørensen 2021). Given this, and given the burgeoning global phenomena of “coronaphobia” and Covid-related increases in mental ill-health (Dubey et al. 2020), more research into the wellbeing and psychosocial needs of people with COPD throughout social distancing is needed (Yohannes 2021).

### Social distancing when isolated to begin with: relational, digital and health inequalities

The so-called “new normal” of the emerging rituals and etiquette of socially distanced interactions are rapidly and often creatively evolving, both face to face and online (Carel et al. 2020; Katila et al. 2020). These preclude the instinct and intuition which have historically informed intercorporeal interactions (Dolezal 2017; Dolezal 2020). For people for whose social relationships involve high levels of human touch through handshakes, embraces or other rituals of status and intimacy, the social distancing of both online and face-to-face encounters can be emotionally draining (Carel et al. 2020) and may lead to uncertainties and ambiguities of social status, positioning and expectation (Katila et al. 2020). People must now discuss and agree in advance whether they will shake hands, how far apart they will stand and which toilets they will use. In addition, face coverings and poor telephone or internet connections can reduce the audibility of voices and obscure the non-verbal communication from expressions which accompany speech, meaning that people must work harder to understand one another (Katila et al. 2020). Given that people with COPD are usually older and less socially advantaged than the population in general (DoH 2011; WHO 2019), it is reasonable to assume that many will be harder of hearing and less able to afford the higher quality electronic devices required for optimal online communication.

Newly emerging online means of interaction and relatedness, for example group video chats rather than travel to events, can provide social opportunities that might not otherwise be accessible to people isolated by limited mobility or other physical impairments (Goli 2020; Rossolatos 2020; Vallee 2020). Social distancing is not necessarily entirely detrimental to social relationships among those who use the internet. However, the growing status and importance of social media and video conferencing as a phenomenologically existing real place by definition excluded those who do not use the internet. Although no specific data exist, it is reasonable to assume the demographic and social profile of people with COPD (DoH 2011; WHO 2019) means that, even in the most technologically developed societies, many cannot access the internet. People with COPD are vulnerable to new ways of becoming lonely, and emerging research suggests their loneliness to be phenomenologically unique (Burlacu et al. 2021).

Social distancing has moved online not only social relationships but professional and public services; people are newly required, for example, to send photo and videos messages of medical symptoms to their doctors and to attend the funerals of their friends via livestream. For those who cannot access online technology due to poverty, remoteness, or lack of infrastructure, for those who struggle to operate digital devices and for those who are uncomfortable with such innovations, this can exacerbate what was already a complicated and multi-faceted “digital divide”, among older adults (Beaunoyer et al. 2020; Ramsetty and Adams 2020). Yoeli and Macnaughton (2021) have found that, for people with COPD, internet use, isolation, and coping are largely socially patterned. Further research is needed, and future research must remain attuned to the specific barriers to social interaction and online care with which people with COPD contend.

### Social distancing as a stigmatised being: fear, mistrust, and scapegoating

By foregrounding the COVID-19 infection risk that individuals pose to one another, social distancing reduces the human body from a social to a physical entity, and then further to a potentially pathological one. The basic premise of social distancing is that all humans are potential carriers of COVID-19 and that all humans bodies are an infection risk both to and from one another (Dolezal 2020; Goli 2020). Inherent within the avoidance of proximity and touch is a fundamental mistrust of other people, and a fear of their bodies. This fear and mistrust is being enacted not only at an individual and interpersonal but a social and global level, attenuating the atmosphere of economic and political and economic uncertainty and crisis the pandemic has brought (Carel et al. 2020).

Plagues and pandemics throughout history have repeatedly demonstrated that people perceived as most vulnerable to the virus have been stigmatised (Logie and Turan 2020), and already-marginalised groups have been scapegoated as potential carriers and spreaders (Jones 2020). Although parallels are frequently drawn between COVID-19 and the global AIDS pandemic of the 1980s and 1990s, the differing means of transmission between the viruses responsible have led to subtly different stigmas. As a blood-borne virus, AIDS specifically requires sexual transmission or intravenous access; as an airborne virus, COVID-19 requires only physical proximity, or an absence of social distancing. Whereas AIDS stigmatised and scapegoated people primarily for their perceived behavioural choices, albeit with overtones of racism (gay sub-cultures, drug users, hypersexualised black men), COVID-19 stigmatises only bodies, and particularly bodies regarded as “deviant” or “other”.

Within the global climate of mistrust and uncertainty, certain groups are finding the experience of having their “different” or “other-ed” bodies stigmatised or shamed is a familiar one—women, queer or disabled people and people of colour (Dolezal 2015a, b)—something which may explain the resurgence of Black Lives Matter protests during the COVID-19 pandemic (Crooks et al. 2021). Given this, given the disproportionate scapegoating of black people during the AIDS pandemic (Logie and Turan 2020), and given the increasingly emotively contested explanations for the increased prevalence and mortality of COVID-19 among people of black and Asian heritage across Europe and North America and people of indigenous heritage across the Americas and Australasia (Blundell et al. 2020), it is likely that the pandemic stigmatisation of people with COPD will become intersectional. People with COPD from disadvantaged racial or ethnic minorities are thereby likely to experience this intensified stigma most acutely (Devakumar et al. 2020). Researchers, like health and social care practitioners, must remain conscious of this.

## Conclusion

This paper has phenomenologically explored three ways that people with COPD—who are already likely to live in poverty, isolation and disadvantage—are at risk of becoming increasingly marginalised, excluded and stigmatised by the social distancing procedures of the COVID-19 pandemic. People with COPD are anxious about contracting the virus and afraid of being denied the medical care they need. COPD tends to affect older people within the most already isolated and marginalised socioeconomic groups. COPD has long been a stigmatised illness, and pandemics are known to intensify existing social stigmas. Therefore, researchers should remain mindful of diversity considerations and of the need to challenge systemic and institutional forms of disadvantage.

Because this paper is intentionally speculative and polemical in nature, it contains few empirical conclusions from which to draw evidence-based assertions. Instead, this paper proposes philosophical and theoretical innovations to stimulate such empirical research. Given the highly unusual and rapidly evolving nature of the COVID-19 pandemic, researchers are increasingly adopting epistemologically more pluralist standpoints, evaluating the theoretical alongside the empirical to inform decision-making processes. The discipline of embodied phenomenology will have a unique contribution to offer in enabling research into the lived experiences of people with COPD during the COVID-19 pandemic.

## References

[CR1] Askov Mousing C, Sørensen D (2021). Living with the risk of being infected: COPD patients’ experiences during the coronavirus pandemic. Journal of Clinical Nursing.

[CR2] Barbisch D, Koenig KL, Shih F-Y (2015). Is there a case for quarantine? Perspectives from SARS to Ebola. Disaster Medicine and Public Health Preparedness.

[CR3] Beaunoyer E, Dupéré S, Guitton MJ (2020). COVID-19 and digital inequalities: Reciprocal impacts and mitigation strategies. Computers in Human Behavior.

[CR4] Blundell, R., M.C. Dias, R. Joyce, et al. 2020. Covid‐19 and inequalities. *Fiscal Studies*. 10.1111/1475-5890.12232 PMC7362053 32836542

[CR5] Burlacu A, Mavrichi I, Crisan-Dabija R (2021). “Celebrating old age”: An obsolete expression during the COVID-19 pandemic? Medical, social, psychological, and religious consequences of home isolation and loneliness among the elderly. Archives of Medical Science: AMS.

[CR6] Carel H, Ratcliffe M, Froese T (2020). Reflecting on experiences of social distancing. The Lancet.

[CR7] Crooks N, Donenberg G, Matthews A (2021). Ethics of research at the intersection of COVID-19 and black lives matter: A call to action. Journal of Medical Ethics.

[CR8] Csordas TJ (1993). Somatic modes of attention. Cultural Anthropology.

[CR9] De Lucia, N., F.P. Ausiello, M. Spisto, et al. 2020. The emotional impact of COVID-19 outbreak in amyotrophic lateral sclerosis patients: Evaluation of depression, anxiety and interoceptive awareness. *Neurological Sciences*, 1–3. 10.1007/s10072-020-04592-2 PMC7359442 32666406

[CR10] Devakumar D, Shannon G, Bhopal SS (2020). Racism and discrimination in COVID-19 responses. The Lancet.

[CR11] DoH (2011). An outcomes strategy for chronic obstructive pulmonary disease (COPD) and asthma in England.

[CR12] Dolezal L (2015). The body and shame: Phenomenology, feminism, and the socially shaped body.

[CR13] Dolezal L (2015). The phenomenology of shame in the clinical encounter. Medicine, Health Care and Philosophy.

[CR14] Dolezal L (2017). The phenomenology of self-presentation: Describing the structures of intercorporeality with Erving Goffman. Phenomenology and the Cognitive Sciences.

[CR15] Dolezal L (2020). Intercorporeality and social distancing: Phenomenological reflections.

[CR16] Dubey S, Biswas P, Ghosh R (2020). Psychosocial impact of COVID-19. Diabetes & Metabolic Syndrome: Clinical Research & Reviews.

[CR17] Ferguson N, Laydon D, Nedjati-Gilani G (2020). Report 9: Impact of non-pharmaceutical interventions (NPIs) to reduce COVID19 mortality and healthcare demand. Imperial College London.

[CR18] Goli F (2020). Connected minds, disconnected bodies; the somatophobic era of Corona. International Journal of Body, Mind and Culture.

[CR19] gov.uk. (2020) *Guidance on shielding and protecting people who are clinically extremely vulnerable from COVID*-*19: Definition of ‘clinically extremely vulnerable’*. https://www.gov.uk/government/publications/guidance-on-shielding-and-protecting-extremely-vulnerable-persons-from-covid-19/guidance-on-shielding-and-protecting-extremely-vulnerable-persons-from-covid-19#definition.

[CR20] Heidegger M (1927). Being and time: A translation of Sein und Zeit (1996).

[CR21] Huremović D, Huremović D (2019). Brief history of pandemics (pandemics throughout history). Psychiatry of pandemics: A mental health response to infection outbreak.

[CR22] Jones DS (2020). History in a crisis—Lessons for Covid-19. New England Journal of Medicine.

[CR23] Katila J, Gan Y, Goodwin MH (2020). Interaction rituals and ‘social distancing’: New haptic trajectories and touching from a distance in the time of COVID-19. Discourse Studies.

[CR24] Leder D (1984). Medicine and paradigms of embodiment. The Journal of Medicine and Philosophy.

[CR25] Logie CH, Turan JM (2020). How do we balance tensions between COVID-19 public health responses and stigma mitigation? Learning from HIV research. AIDS and Behavior.

[CR26] Mackowiak PA, Sehdev PS (2002). The origin of quarantine. Clinical Infectious Diseases.

[CR27] Macnaughton J (2020). Making breath visible: Reflections on relations between bodies, breath and world in the critical medical humanities. Body & Society.

[CR28] Markel H, Lipman HB, Navarro JA (2007). Nonpharmaceutical interventions implemented by US cities during the 1918-1919 influenza pandemic. JAMA.

[CR29] McAuley H, Hadley K, Elneima O (2021). COPD in the time of COVID-19: An analysis of acute exacerbations and reported behavioural changes in patients with COPD. ERJ Open Research.

[CR30] Merleau-Ponty M (1968). The visible and the invisible: Followed by working notes.

[CR31] nhs.uk. 2020. *Who’s at higher risk from coronavirus*. https://www.nhs.uk/conditions/coronavirus-covid-19/people-at-higher-risk/whos-at-higher-risk-from-coronavirus/.

[CR32] Philip, K.E.J., B. Lonergan, A. Cumella, et al. 2020. COVID-19 related concerns of people with long-term respiratory conditions: A qualitative study. *medRxiv*. 10.1186/s12890-020-01363-9 PMC7724437 33298023

[CR33] Ramsetty A, Adams C (2020). Impact of the digital divide in the age of COVID-19. Journal of the American Medical Informatics Association.

[CR34] Rossolatos, G. 2020. So near, so far, so what is social distancing? A fundamental ontological account of a mobile place brand. SSRN 3618795.

[CR35] Vallee M (2020). Doing nothing does something: Embodiment and data in the COVID-19 pandemic. Big Data & Society.

[CR36] Vestbo J, Hurd SS, Agustí AG (2013). Global strategy for the diagnosis, management, and prevention of chronic obstructive pulmonary disease: GOLD executive summary. American Journal of Respiratory and Critical Care Medicine.

[CR37] Wallis, P. 2006. A dreadful heritage: Interpreting epidemic disease at Eyam, 1666–2000. *History Workshop Journal.* Oxford University Press, 31–56. 10.1093/hwj/dbi060 16967576

[CR38] WHO. 2019. *Chronic Obstructive Pulmonary Disease (COPD)*. https://www.who.int/respiratory/copd/en/.

[CR39] Williams T, Carel H, Aho K (2018). Breathlessness: From bodily symptom to existential experience. Existential medicine.

[CR40] Yoeli, H., and J. Macnaughton. 2021. ‘You just keep breathing, don’t you?’ Lived experiences of COPD and resilience during the Covid-19 pandemic *Aging & Society (forthcoming)*.

[CR41] Yohannes AM (2021). COPD patients in a COVID-19 society: Depression and anxiety.

